# AI-assisted case-based learning and flipped classroom to improve clinical decision-making: a randomized controlled trial in reproductive medicine

**DOI:** 10.1080/10872981.2026.2670047

**Published:** 2026-05-27

**Authors:** Qi Wang, Cong Hu, Yiyang Li, Ting Zhang, Songling Zhang

**Affiliations:** a Prenatal Diagnosis Center, Reproductive Medicine Center, The First Hospital of Jilin University, Changchun, Jilin, People's Republic of China; b Obstetrics and Gynecology Center, The First Hospital of Jilin University, Changchun, Jilin, People's Republic of China

**Keywords:** Assisted reproductive medicine, artificial intelligence, case-based learning, flipped classroom, clinical decision-making, resident training

## Abstract

**Background:**

Efficient training of reproductive medicine clinicians is critical in the context of declining global fertility and increasing infertility. Traditional lecture‑based instruction often fails to sufficiently develop clinical decision‑making skills within limited residency rotations. Innovative strategies that integrate artificial intelligence (AI) with case‑based learning (CBL) and flipped classroom (FC) formats may enhance clinical reasoning, however rigorous evidence in reproductive medicine education remains limited.

**Methods:**

We conducted a randomized controlled trial involving 50 obstetrics and gynecology residents at the First Hospital of Jilin University. Participants were randomly assigned to an AI‑assisted CBL+FC group or a traditional lecture control group. The AI‑assisted CBL+FC group completed pre‑class interactive case work with virtual standardized patients on the DoctorU platform and case analyses on the Superstar Learning platform, followed by interactive in‑class discussions. Primary outcomes included post‑course theoretical knowledge tests, Mini‑Clinical Evaluation Exercise (Mini‑CEX), and Objective Structured Clinical Examination (OSCE) scores. Secondary outcomes assessed learner motivation, clinical thinking, self‑directed learning, and perceived course effectiveness using a 5‑point Likert scale.

**Results:**

Baseline characteristics were comparable between groups. After the intervention, the AI‑assisted CBL+FC group achieved significantly higher theoretical test scores than the control group. The AI‑assisted CBL+FC group also demonstrated superior overall clinical competence in Mini‑CEX assessments and higher OSCE total scores. Participants in the AI‑assisted CBL+FC group reported greater improvements in learning motivation, clinical reasoning, self‑directed learning, and perceived course effectiveness.

**Conclusions:**

The AI‑assisted CBL+FC instructional model significantly enhances theoretical knowledge, clinical decision‑making skills, and learner engagement among reproductive medicine residents. This blended learning model offers an efficacious and generalizable methodology for training practitioners to address the evolving clinical requirements within contemporary fertility care.

## Introduction

The global convergence of declining birth rates and increasing infertility has brought the issues of reproductive health and demographic sustainability to the forefront of public health agendas [1,2]. Addressing these demographic dynamics requires that healthcare infrastructures elevate the efficient training of skilled reproductive specialists to a top public health priority, thus ensuring the delivery of high quality care essential for optimal fertility outcomes.

The competency framework for reproductive medicine physicians extends far beyond the boundaries of general gynaecologist. In addition to expertise in gynaecologist, obstetrics, endocrinology, and embryology, reproductive physicians must possess a robust foundation in immunology, genetics, and ultrasound medicine, and maintain a nuanced understanding of medical ethics pertaining to assisted reproduction. These interdisciplinary skills are essential not only to deliver effective assisted reproductive technology (ART) procedures but also to ensure safety, ethical integrity, and the pursuit of favourable genetic and developmental outcomes in newborns [3].

According to our assessment of educational needs within the First Hospital of Jilin University, residents prioritised the integration of complex clinical scenarios into their training to better facilitate decision-making in ART (Table S1). Traditional training models, however, often regard reproductive medicine as a minor subspecialty within gynaecologist. This approach results in fragmented learning experiences and prolonged clinical competency cycles, which are misaligned with the urgency of current fertility challenges. Innovative, integrated, and efficient educational strategies are therefore urgently needed. Case-based learning (CBL) offers a means to bridge the gap between theoretical knowledge and clinical application by placing learners in authentic decision-making scenarios [4]. Within reproductive medicine, CBL fosters clinical reasoning and enhances evidence-based decision-making. Nonetheless, large-scale adoption of CBL is hindered by limited classroom time and the challenge of delivering personalised instructional support to all residents [5,6]. Many educators, accustomed to traditional didactic teaching, may find it challenging to manage the dynamic nature of case-based discussions or may lack specific training in interactive facilitation. Therefore, to ensure that residents fully acquire the necessary professional capabilities, it is essential to not only extend the rotation duration but also to focus on faculty development and explore more cost-effective, instructor-friendly training methods. Integrating CBL with the flipped classroom (FC) model provides a promising pedagogical innovation. By shifting foundational knowledge acquisition to pre-class activities, the FC format frees in-class time for collaborative case discussions and complex decision-making simulations [7]. This hybrid CBL + FC model maximises the value of in-class learning but remains constrained by limited personalised guidance and insufficiently diverse learning resources [8]. Advances in artificial intelligence (AI) also present new opportunities to address these gaps [9,10]. Through large-scale data analytic and machine learning, AI can deliver tailored learning pathways, simulate realistic clinical scenarios, and optimise educational efficiency [11]. In ART procedures, AI has already demonstrated clinical value in ovarian stimulate protocol optimisation and embryo selection [12]. However, few rigorous empirical studies have systematically examined AI-assisted CBL + FC approaches, particularly in relation to the development of interdisciplinary clinical decision-making competence among reproductive medicine trainers within accelerated, time-limited training frameworks.

To address the urgent need for enhancing obstetrics and gynaecologist residents’ knowledge acquisition, encompassing ethical, immunological, and genetic considerations and clinical decision-making capabilities in reproductive medicine, we implemented a randomised controlled trial to evaluate an AI-assisted hybrid CBL + FC instructional model. In contrast to conventional AI-assisted instructional models, our approach implements a dynamic adaptation to individual learning trajectories. By integrating with FC framework, we facilitate targeted pedagogical interventions through intensive teacher-student discourse. This synergistic approach transcends basic knowledge acquisition, fostering the higher-order clinical decision-making competencies indispensable for the field of reproductive medicine. We further anticipate higher engagement and satisfaction among participants, supporting the adoption of this innovative approach to meet the growing global demand for highly skilled reproductive specialists.

## Materials and methods

### Study design and participants

This randomised controlled trial was conducted between September 2025 and December 2025, in the Department of Obstetrics and gynaecologist, First Hospital of Jilin University. Eligible participants were resident physicians who had completed theoretical and clinical rotations in physiology, histology and embryology, internal medicine, surgery, obstetrics and gynaecologist, and paediatrics, in accordance with the national standardised residency training curriculum. All participants were actively enroled in the standard residency programme in Obstetrics and gynaecologist at the time of study initiation. Participants were randomly allocated by lot drawing into either an AI assistied CBL + FC group or a control group.

### Inclusion and exclusion criteria

Inclusion Criteria were as follows, ①Completion of theoretical coursework (such as, physiology, histology and embryology, internal medicine, surgery, Obstetrics and Gynaecology, and paediatrics). ②Currently participating in standard residency training in the Department of Obstetrics and gynaecologist. ③Ability to adhere to the assigned instructional model and complete the teaching programme. Exclusion Criteria included, ① Prior rotation in reproductive medicine. ②Withdrawal or discontinuation from the study for personal reasons.

### Teaching materials

***Superstar learning.*** The *Superstar Learning System* (https://www.chaoxing.com/) is a mobile-based-learning management platform designed by Beijing Shijichaoxing Information Technology Development Co., Ltd, built upon micro-service architecture to support blended and mobile learning environments [13]. It integrates multimedia teaching resources, knowledge dissemination, and administrative functions.

***DoctorU.*** A human–machine collaborative clinical reasoning training software integrating AI-powered virtual standard patients (SPs) and digital faculty was utilised. This system enabled free-text history taking, simulated physical examination, laboratory and ancillary test ordering, and provision of corresponding results. Learners addressed patient concerns on diagnosis, management, prognosis, and health outcomes, allowing assessment of communication skills and professionalism. The model utilised Retrieval-Augmented Generation principles by integrating a specialised ART database. Inter-rater reliability and content validity were confirmed by two senior experts prior to implementation, ensuring alignment with standardised clinical practice.

***Mini-Clinical evaluation exercise (Mini-CEX).*** Mini-CEX is a tool used to assess clinical skills in medical education, widely applied in the evaluation of clinical competence among medical students and residents. It is a structured, face-to-face assessment method designed to evaluate the performance of medical students or residents in real clinical settings, with a particular focus on patient interaction, clinical decision-making, communication skills, and professional behaviour.The Mini-CEX scoring system uses a 9-point scale, with seven criteria: medical interviewing skills, physical examination skills, humanistic qualities/professionalism, clinical judgement, counselling skills, organisation efficiency an overall clinical competence. Scores of 1−3 indicate unsatisfactory performance, 4-6 indicate satisfactory performance, and 7–9 indicate excellent performance.

***Objective structured clinical examination (OSCE)*** The OSCE assessment encompassed three domains: medical history collection, physical examination, and report analysis. Each item has a maximum score of 20 points, with a total possible score of 60 points.

### Intervention protocol

All sessions were taught by the same instructor, using the same syllabus covering assisted reproductive medicine topics, including fundamentals of reproductive endocrinology; selection of ovulation induction protocols in polycystic ovarian syndrome; infertility diagnosis and management; recurrent miscarriage management; and clinical case studies in assisted reproductive technologies. Instruction encompassed medical history collection, physical examination, diagnosis, differential diagnosis, and treatment planning. Total instructional time was six sessions for each group.

Participants in the control group were assigned individual pre-class preparation, which was followed by a traditional didactic lecture where the instructor provided detailed, pre-scripted explanations. Conversely, participants in the AI assisted CBL + FC group were instructed to interact with virtual SPs, generated using real patient data on the *DoctorU* platform, as part of their pre-class work and case analysis tasks completed via Superstar Learning. The learning progress analysis of *Superstar Learning* provides a clear view of each student's mastery of every individual knowledge point (Figure S1). Upon completion, a full medical record and related pre-class learning points were released for review. Subsequently, learners identified persistent questions for small-group discussions and presentations. Key questions, collated by a student representative, were submitted to the instructor. The session concluded with the instructor clarifying unresolved issues, correcting misunderstandings, and delivering focused teaching on areas of difficulty. Two independent examiners, who were not involved in the teaching process, were responsible for scoring the Mini-CEX and OSCE assessments. To ensure objectivity, the two examiners were blinded to the students’ group assignments and received prior training to standardise the scoring criteria.

### Outcome measures

#### Primary outcomes

Before class, via the Superstar Learning platform, both the AI-assisted CBL + FC group and the control group completed the same pre-class test, and pre-class SP interview scores were recorded for the AI-assisted CBL + FC group. Post-course assessment included: ①Theoretical Knowledge Test (maximum score: 100 points) covering key elements of ART theory. ②Comprehensive Skills Assessment—combining learning progress analysis from *Superstar Learning*, the Mini-CEX assessmen and an OSCE format.

#### Secondary outcomes

Upon completion of the course, in addition to assessing knowledge acquisition and clinical decision-making skills, both groups completed an identical questionnaire evaluating five additional dimensions: motivation for learning, improvement in clinical thinking, enhancement of teamwork awareness, self-directed learning ability, and perceived course effectiveness. Responses were rated on a 5-point Likert scale (1 = very dissatisfied, 5 = very satisfied). Additionally, a post-hoc survey to quantify the pre- and post- learning time for both groups to address this concern was also conducted.

### Statistical analysis

Statistical analysis was conducted using SPSS 27.0 software. Quantitative data are presented as mean ± standard deviation ( $\bar{x}$ ± SD) and analysed using t-tests. Categorical data are presented as rates and analysed using chi-square (χ²) tests. A *P* value of less than 0.05 was considered statistically significant.

## Results

### Base character of participates

**Figure 1.**
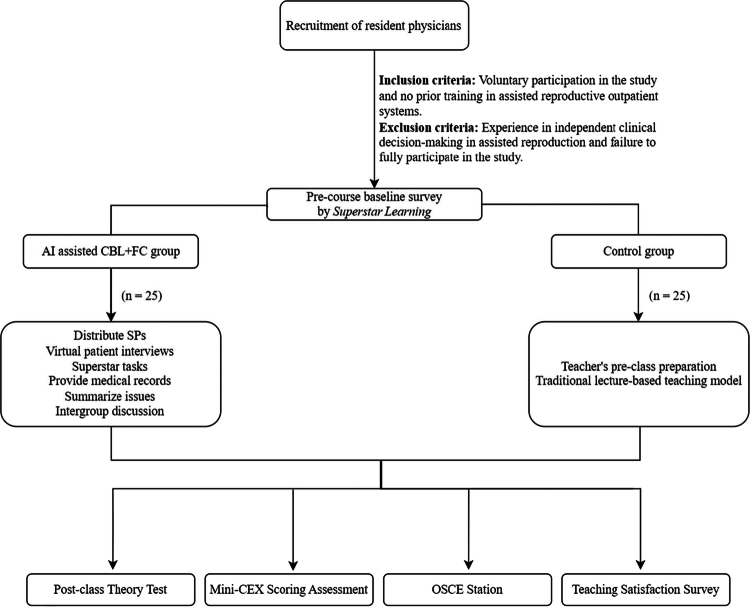
Study design and flow chart.

Fifty resident physicians who met the inclusion criteria were randomly assigned to two groups via lottery, with 25 participants in each group (*n* = 25 each) (Figure 1). Baseline characteristics, including age, pre-course grade point average (GPA), pre-course test scores, total duration of internship, and sex were compared between the two groups (Table 1). There was no statistically significant differences (*P* > 0.05), confirming baseline comparability.

**Table 1.** Comparison of baseline characteristics between the two groups.

	Control group (Mean ± SD)	AI assisted CBL + FC group (Mean ± SD)
Age (years)	24.60 ± 1.12	24.56 ± 1.19
Pre- course GPA	2.94 ± 0.20	2.98 ± 0,29
Pre-course test	63.80 ± 7.54	63.20 ± 7.34
Duration of internship Sex (%)	1.54 ± 0.89 Male(0.12) Female(0.88)	1.78 ± 0.74 Male(0.08) Female(0.92)

*P* > 0.05.

### Post-class theoretical scores

**Figure 2.**
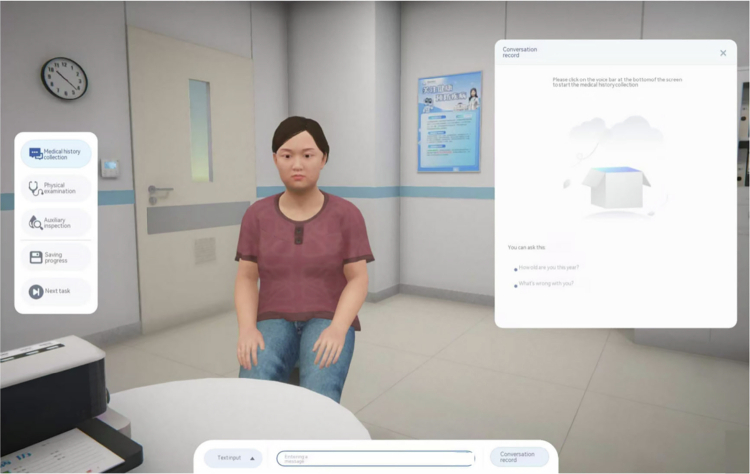
DoctorU software screenshot.

**Figure 3.**
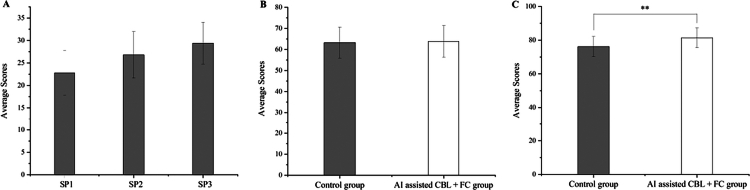
Post-class theoretical scores. (A): Test scores of the AI assisted CBL + FC group after three SPs consultations. (B&C):Comparison chart of average scores for pre-and post-class tests. **P＜0.01.

The two groups received different instructional methods, one group received traditional teaching, and the other received AI-assisted CBL + FC. In the traditional teaching group, we presented the cases SP1, SP2, and SP3 during case lectures. In the AI-assisted CBL + FC group, through the Doctor U platform, students engaged in dialogue with SPs for history taking, ordered appropriate ancillary tests, and made diagnoses and differential diagnoses, with the Doctor U platform automatically scoring their performance (Figure 2). A progressive increase was observed in the comprehensiveness of students' patient inquiries from SP1 to SP3. Statistical analysis confirmed that the improvement was significant between SP1 and SP2 (*P* < 0.01) and highly significant between SP1 and SP3 (*P* < 0.001) (Figure 3A).

To evaluate knowledge retention, a post-course examination was administered two weeks after the completion of the teaching intervention. Both groups demonstrated significant improvement from their pre-course baselines (both *P* < 0.05). Moreover, the AI-assisted CBL + FC group significantly outperformed the control group on the post-test (mean score: 81.4 vs. 76.2 points; *P* < 0.01) as shown in Figure 3B and C.

### Mini-CEX assessment scoring

**Figure 4.**
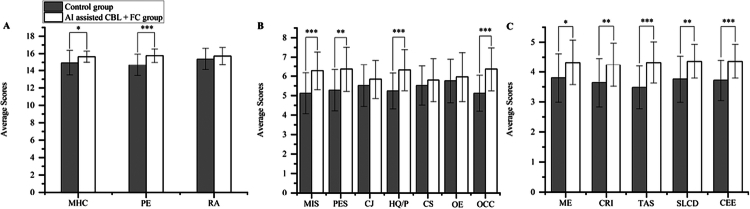
Overall skills and teaching satisfaction scores. (A) The OSCE stations scores between the two groups of students. MHC: Medical History Collection; PE: Physical Examination; RA: Report Analysis. (B) Mini-CEX scores between the two groups of students. MIS: Medical Interviewing Skills; PES: Physical Examination Skills; CJ: Clinical Judgement; HQ/P: Humanistic Qualities/Professionalism; CS: Counselling Skills; OE: Organisation Efficiency; OCC: Overall Clinical Competence. (C) Comparison of teaching satisfaction between two groups. ME: Motivation Enhancement; CRI: Clinical Reasoning Improvement; TAS: Teamwork Awareness Strengthening; SLCD: Self-directed Learning Capacity Development; CEE: Course Effectiveness Evaluation. *P＜0.05, **P＜0.01, ***P＜0.001.

Subsequent to the knowledge test, clinical skills were evaluated via the Mini-CEX. Examiners assessed students using a stable infertile patient case, scores the students based on their performance, completing all ratings within 40 minutes. The AI-assisted CBL + FC group showed statistically significant enhancements relative to the control group. Superior performance was observed in medical interviewing, physical examination, humanistic qualities, professionalism, and overall clinical competence (Table 2 and Figure 4B).

**Table 2.** Mini-CEX scores between the two groups of students.

	Control group (Mean ± SD)	AI assisted CBL + FC group (Mean ± SD)	*P* value
Medical Interviewing Skills	5.12 ± 1.05	6.28 ± 0.98	0.0002***
Physical Examination Skills	5.28 ± 1.06	6.36 ± 1.15	0.001**
Clinical Judgement	5.52 ± 1.08	5.84 ± 0.99	0.281
Humanistic Qualities/Professionalism	5.24 ± 0.93	6.32 ± 1.07	0.0004***
Counselling Skills	5.52 ± 1.00	5.80 ± 1.12	0.356
Organisation Efficiency	5.76 ± 1.13	5.96 ± 1.27	0.560
Overall Clinical Competence	5.12 ± 0.93	6.36 ± 1.11	0.00009***

*P＜0.05, **P＜0.01, ***P＜0.001.

### OSCE station scoring

Objective clinical performance was further assessed via an OSCE conducted within one week post-course. The AI-assisted CBL + FC group achieved significantly higher mean scores than the control group across all three assessment stations (Figure 4A, Table 3).

**Table 3.** The OSCE stations scores between the two groups of students.

	Control group (Mean ± SD)	AI assisted CBL + FC group (Mean ± SD)	*P* value
Medical History Collection	14.92 ± 1.44	15.64 ± 0.64	0.027*
Physical Examination	14.68 ± 1.25	15.76 ± 0.78	0.0006***
Report Analysis	15.36 ± 1.25	15.72 ± 0.98	0.264
Total	44.96 ± 2.26	47.12 ± 1.62	0.0003***

*P＜0.05, **P＜0.001

### Teaching satisfaction scoring

Beyond objective performance, subjective feedback revealed a strong preference for the integrated teaching model. Students in the AI-assisted CBL + FC group reported significantly higher levels of satisfaction, offering more affirmative evaluations of the learning approach compared to the control group (Figure 4C).

### Time on task

Although the AI-assisted CBL + FC model required more intensive pre-class preparation compared to the traditional model, which was offset by a significant reduction in post-class review time. The total cumulative study time remained comparable between the two cohorts (Supplementary Table S2).

## Discussion

This study systematically integrated an AI-assisted CBL approach with the FC model to examine its utility in strengthening clinical decision-making competencies in assisted reproductive medicine. Our findings demonstrated that, compared with conventional lecture-based training, the AI-assisted CBL + FC methodology significantly improved residents' performance in core clinical domains, including medical history acquisition, diagnostic reasoning, and formulation of treatment plans, alongside measurable gains in overall learning efficiency. The instructional architecture of this model is predicated on strategic redistribution of cognitive workload: foundational content acquisition is transitioned to the pre-class stage via targeted e-learning and virtual SP engagement [14]. This allows classroom sessions to be dedicated to high-order learning activities, such as synthesis of complex clinical data, inter-group debates, and simulation-based diagnostic and therapeutic decision-making [15]. The total cumulative study time remained comparable between the two cohorts. This time-redistribution effect suggests that AI-assisted learning enhances initial acquisition and retention, allowing for a more efficient mastery of clinical competencies without increasing the total workload, which maybe is the crucial factor for the scalability of this pedagogical approach. By requiring participants to assume primary responsibility as clinical decision-makers in realistic case scenarios, this framework accelerates the translation of theoretical knowledge into functional clinical competence. The incorporation of AI enriches this methodology by enabling dynamic case generation, adaptive questioning, and automated feedback. These features are widely recognised as beneficial in educational theory, our findings indicate that AI-assisted learners demonstrated statistically significant improvements in post-course exam scores, Mini-CEX performance, and OSCE results compared to the control group. These empirical results confirm that the AI system’s adaptive feedback and personalised learning paths directly enhanced clinical decision-making and knowledge retention. In contrast, the broader theoretical advantages of AI, such as dynamic case generation and real-time feedback, remain hypothetical mechanisms that require further investigation to establish their causal impact on learner performance. AI-driven analytics facilitate granular tracking of individual learning trajectories, allowing instructors to identify persistent gaps and tailor reinforcement accordingly.

In the context of reproductive medicine, a discipline inherently reliant on nuanced, individualised patient management integrating historical data, endocrine profiles, imaging, and laboratory findings, such an approach fosters the development of a comprehensive and systematic cognitive framework [16]. Moreover, the integration of AI-mediated simulation provides an environment where complex clinical decision-making can be practiced repeatedly with consistent fidelity, a factor known to enhance procedural confidence and diagnostic accuracy. Reproductive specialists often need to integrate multiple sources of information, such as patient history and endocrine levels, to develop precise and personalised treatment plans [17,18]. This aligns with mounting evidence from broader medical education literature suggesting that AI-supported virtual patient platforms can serve as sustainable, scalable tools for immersive experiential learning. The AI-assisted CBL + FC teaching model offers a viable path for skill development and improvement. Through AI's intelligent analysis of case data and laboratory parameters, students can develop a more profound understanding of the subtle interactions among various variables, thus establishing a comprehensive and systematic clinical cognition framework. Moreover, the incorporation of AI systems into the educational platform enables instructors to assess students' learning progress and offer personalised feedback. By specifically strengthening each student's weak points, their self-directed learning ability and classroom participation are enhanced [19]. Compared to traditional teaching methods or the mere combination of CBL + FC model, the introduction of AI-assisted teaching strategies is more prominent in addressing issues such as insufficient personalised guidance and the scarcity of teaching resources [20,21]. It is evident that AI not only aids in enhancing individual clinical capabilities but also provides a data-driven approach for improving medical education. It offers a reference model for the design of future teaching programmes [22].

Despite its strengths, this study has several limitations. First, we did not quantitatively measure the exact time students spent on pre-class preparation, while faculty estimations indicated a comparable workload across groups during pilot testing. Future studies should aim to quantify the time-on-task for each model to better understand the potential differences in learning time between the two groups and how it may impact learning outcomes. Second, the inherent constraints of current AI models must be acknowledged, particularly regarding the generalisability of training data and the risk of clinical inaccuracies. The successful integration of virtual SPs requires comprehensive faculty training to transition educators from traditional lecturers to AI-literate facilitators. In this initial phase, we intentionally utilised standardise cases to mitigate these risks. In addition, the study design was a single-centre trial with a limited sample size, which may limit the generalisability of our findings. The relatively short follow-up period also poses challenges in assessing the long-term impact of AI-assisted learning on clinical decision-making. Multi-centre, longitudinal trials with larger sample sizes are needed to further substantiate our findings. Regarding the contribution of AI versus the FC component, while both were integrated in this study, their relative effects on the observed learning outcomes remain unclear. The AI system played a significant role in personalising learning and providing dynamic case generation, while the FC model allowed for collaborative, high-order discussions. Future research should aim to disentangle these two components to better understand their individual contributions to improving clinical decision-making and knowledge retention. The exploration of advanced generative AI for constructing evolving case libraries, combined with precision feedback systems driven by real-time analytics, could further elevate the pedagogical impact. Importantly, such developments may redefine the instructional paradigm in reproductive medicine, shifting from a predominantly knowledge-transmission model toward the targeted cultivation of clinical decision-making agility, ethical reasoning, and interdisciplinary diagnostic integration.

In conclusion, AI-assisted CBL integrated with the FC model demonstrates clear advantages in enhancing clinical decision-making capabilities, learner engagement, and educational efficiency among physicians in assisted reproductive medicine. This blended, technology-enabled strategy not only addresses key limitations of conventional teaching, but also offers a scalable blueprint for innovation across other medical specialties. With continual refinement and broader implementation, this approach holds considerable promise for shaping the next generation of reproductive medicine specialists, ensuring they are equipped with the cognitive flexibility, ethical awareness, and interdisciplinary expertise required to meet the complex demands of contemporary fertility care.

## Supplementary Material

Supplementary Material Supplementary Materials.docx

Supplementary Material CONSORT 2025 checklist.docx

## Data Availability

The datasets during and/or analysed during the current study are available from the corresponding author on reasonable request. The trial protocol and statistical analysis plan are also available upon request.
