# Supplementary material for: AI-assisted case-based learning and flipped classroom to improve clinical decision-making: a randomized controlled trial in reproductive medicine
Source: Med Educ Online. 2026 May 27;31(1):2670047. doi: 10.1080/10872981.2026.2670047 (PMC13218306; doi:10.1080/10872981.2026.2670047)
Supplement: Supplementary Material — s.docx [file ZMEO_A_2670047_SM5963.docx]

Supplementary Materials

**Supplementary figure1**


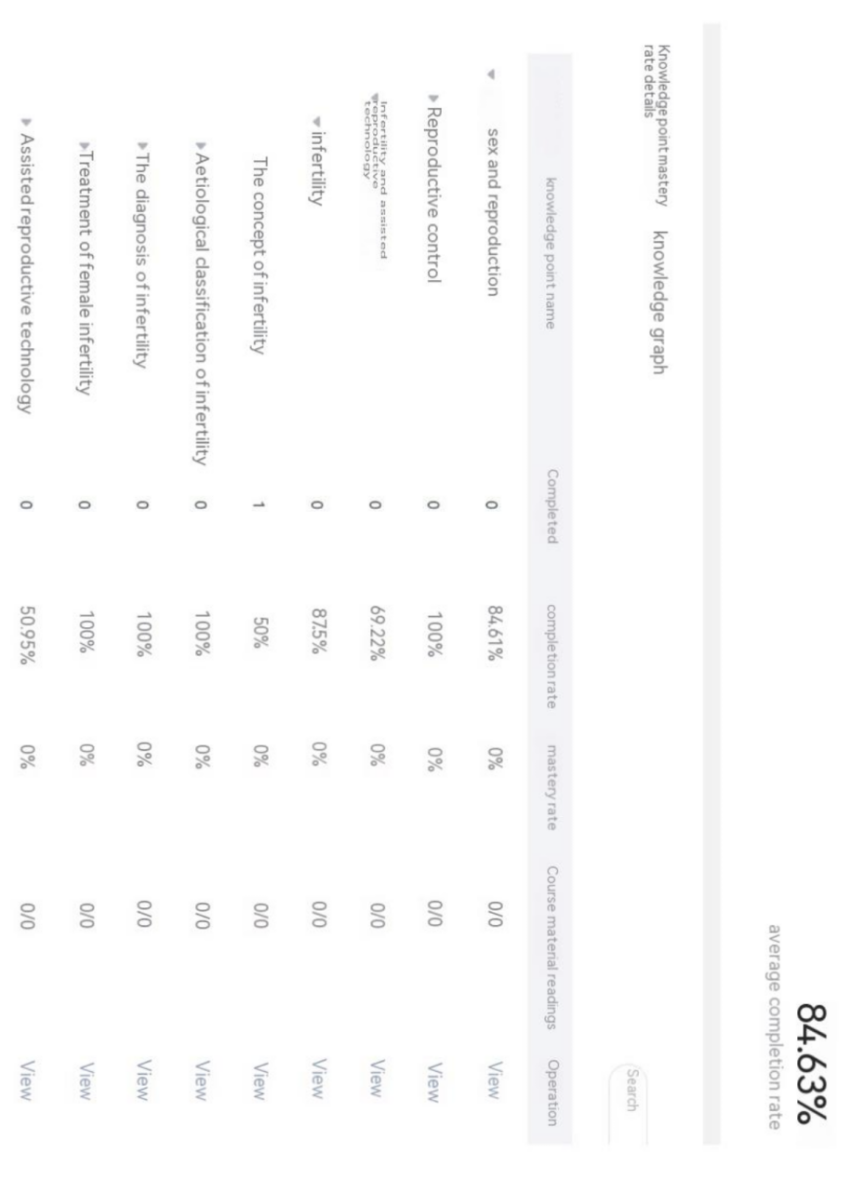


**Figure S1** Learning Progress Analysis in Superstar Learning

**Supplementary tables**

**Table S1** Survey on resident physicians' needs for clinical decision-making skills in assisted reproduction

| Item | Percentage (%) |
| --- | --- |
| Increase case analysis and training related to clinical decision-making | 70.21 |
| Increase virtual patient simulation training | 58.42 |
| Strengthen self-directed learning capacity | 52.13 |
| More interactive flipped classroom teaching | 45.67 |
| Stronger clinical skills training and evaluation | 38.93 |

**Table S2** Comparison of pre-class preparation time, post-class review time, and total learning time between the two groups

| **Time Variable** | **Control group**  **(Mean ± SD, minutes)** | **AI assisted CBL + FC group**  **(Mean ± SD, minutes)** | ***P* value** |
| --- | --- | --- | --- |
| Pre-class Preparation Time | \| 45.96 ± 10.3 \| \| --- \| | 66.36 ± 2.96 | < 0.001 |
| Post-class Review Time | 75.24 ± 15.2 | 54.88 ± 1.74 | < 0.001 |
| Total Learning Time | \| 121.28 ± 2.07 \| \| \| --- \| --- \| \|  \| | 120.84 ± 2.82 | \| 0.533 \| \| --- \| |
